# Biomarkers of Lung Injury in Cardiothoracic Surgery

**DOI:** 10.1155/2015/472360

**Published:** 2015-03-17

**Authors:** Gerwin Erik Engels, Willem van Oeveren

**Affiliations:** ^1^HaemoScan BV, 9723 JC Groningen, Netherlands; ^2^Department of Cardiothoracic Surgery, University of Groningen, University Medical Center Groningen, 9713 GZ Groningen, Netherlands

## Abstract

Diagnosis of pulmonary dysfunction is currently almost entirely based on a vast series of physiological changes, but comprehensive research is focused on determining biomarkers for early diagnosis of pulmonary dysfunction. Here we discuss the use of biomarkers of lung injury in cardiothoracic surgery and their ability to detect subtle pulmonary dysfunction in the perioperative period. Degranulation products of neutrophils are often used as biomarker since they have detrimental effects on the pulmonary tissue by themselves. However, these substances are not lung specific. Lung epithelium specific proteins offer more specificity and slowly find their way into clinical studies.

## 1. Introduction

Cardiothoracic surgery, defined as surgery on the heart and/or the lungs, has been performed since the 1950s and coronary artery bypass graft surgery, for instance, has increased to 415.000 procedures a year in the US (US Hospital Discharge Survey, 2009). From the beginning these procedures were associated with postoperative pulmonary complications [1]. These complications can partly be attributed to the unique aspects of cardiothoracic surgery, such as the sternotomy, cardioplegia, and use of cardiopulmonary bypass (CPB) with the exclusion of lung circulation. Despite continuous improvements in materials and surgical techniques, cardiothoracic surgery still causes lung injury, dysfunction, and delay of pulmonary recovery [2].

Lung dysfunction is common after cardiothoracic surgery [2] and varies between hypoxemia in all patients [3] and acute respiratory distress syndrome (ARDS) in 2% of the patients [4]. Lung dysfunction by itself may not influence the postoperative course of a patient; only when lung dysfunction evolves into a lung complication it becomes clinically relevant. Common pulmonary complications following cardiothoracic surgery are pleural effusion (38%) [5], atelectasis (20%) [6], phrenic nerve paralysis (32%) [7], and pneumonia (5%) [8]. Given the high incidence of pulmonary complications, it is important to monitor the onset and course of postoperative lung dysfunction.

Currently there is no gold standard for quantifying postoperative lung injury and dysfunction. Reported are a vast series of physiological changes (alveolar-arterial oxygen pressure difference, intrapulmonary shunt, degree of pulmonary edema, pulmonary compliance, and pulmonary vascular resistance) and measurement of inflammatory markers such as neutrophil elastase, myeloperoxidase, and interleukins [2]. Lung dysfunction, in the form of ARDS, is determined by specific diagnostic criteria recently revised according to the “Berlin criteria” [9]. Acute respiratory distress syndrome is characterized by the acute onset of lung injury within one week of a known clinical insult, bilateral opacities on chest imaging, respiratory failure not fully explained by cardiac failure or fluid overload, and decreased arterial PaO_2_/FiO_2_ ratio. Furthermore, ARDS can be divided into “mild” (PaO_2_/FiO_2_ ratio: 201–300 mmHg), “moderate” (PaO_2_/FiO_2_ ratio: 101–200 mmHg), and “severe” (PaO_2_/FiO_2_ ratio: ≤100 mmHg). Mild ARDS is comparable to the previous acute lung injury (ALI) definition of the American-European Consensus Conference [10].

Biomarkers, whether in serum, urine, or exhaled breath, have found limited use for identifying and quantifying postoperative lung injury. In this review, we focus on the field of cardiothoracic surgery, where serum biomarkers of lung injury are being used as a surrogate marker for (sub)clinical lung injury.

## 2. Postoperative Pulmonary Dysfunction

A distinction between pulmonary dysfunction and pulmonary complications following cardiothoracic surgery should be made [11], where pulmonary dysfunction refers to alterations in pulmonary function, such as shallow respiration and hypoxemia, and where pulmonary complications also require associated clinical findings such as atelectasis and chest radiographic infiltrates.

## 3. Biomarkers

Biomarkers are measurable parameters that reflect the state of a biological process. An often-cited definition of a biomarker is as follows: “a characteristic that is objectively measured and evaluated as an indicator of normal biological processes, pathogenic processes, or pharmacologic responses to a therapeutic intervention [12].” Biomarkers are used to screen for, diagnose, or monitor disease and are also used to assess a therapeutic response [13]. The term biomarker is typically used for molecular biomarkers measured in blood, which is also the application we are focusing on in this review.

In cardiothoracic surgery research, biomarkers can serve as a surrogate endpoint for evaluating new procedures and/or medical equipment, giving more insight into a cellular level. Ideally biomarkers have features such as high sensitivity, high specificity, known reference values, and good predictive values. In case of a surrogate endpoint, biomarker values should have a good correlation with a clinical endpoint. However, when taking serial measurements during cardiothoracic surgery each patient serves as his or her own control making features such as high sensitivity and specificity less important.

Since sample sizes are usually limited in cardiothoracic surgery research, a biomarker could reveal a difference between study groups, whereas differences would not appear when clinical endpoints are considered. Another benefit of biomarkers is that they could give insight into the mechanism of disease, since the measurement is closer to the exposure/intervention of interest and it may be easier to relate causally than more distant clinical events. Being more sensitive, biomarkers could indicate subclinical benefits in a pilot study, supporting larger clinical studies afterwards.

## 4. Classification

The pathophysiology of postoperative lung injury is characterized by injury to the alveolar-capillary membrane, inflammation, increased permeability, and pulmonary edema [14]. Accordingly, biomarkers of lung injury can be classified as such. However, in this review we have focused on biomarkers originating from the alveolar compartment which can be measured in the circulation. We have organized the biomarkers using their cell of origin.

## 5. Polymorphonuclear Neutrophils

Polymorphonuclear neutrophils (PMNs) play an important role in lung injury following cardiothoracic surgery, as their release products can be detrimental to lung tissue. Moreover, the lungs harbour a number of leukocytes equal to or even more than the number of leukocytes present in the systemic circulation [15]. This is usually referred to as the* marginated pool*, which acts as a natural reservoir of leukocytes and is in dynamic equilibrium with the systemic circulation.

### 5.1. Neutrophil Elastase

**Figure 1:**
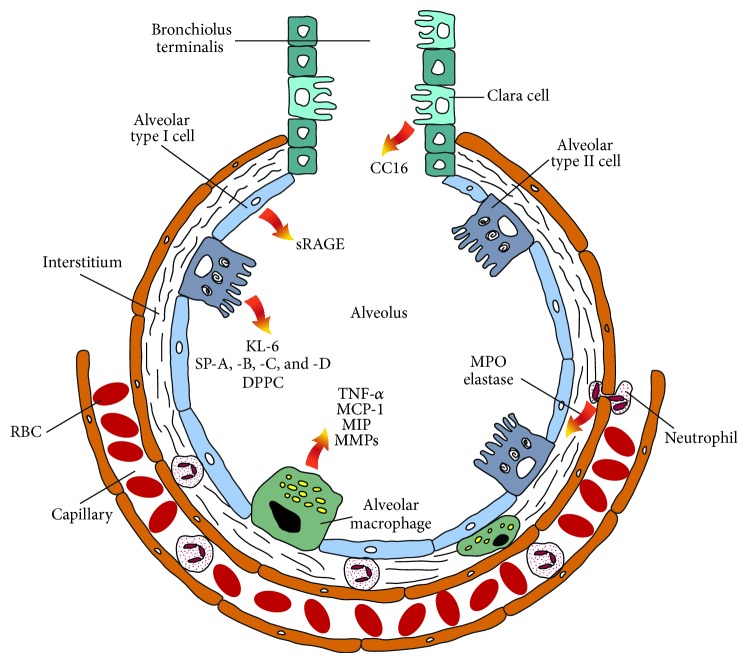
Schematic representation of an alveolus with its various cell types and their secretion products which may serve as lung injury biomarkers.

Upon an inflammatory response PMNs degranulate and release an abundance of cytotoxic substances, such as serine proteases, metalloproteases, peroxidases, and reactive oxygen species (Figure 1) [16]. One of the serine proteases is neutrophil elastase (NE). Besides being a biomarker, NE is an enzyme that has an active role in the development of lung injury. It can degrade components of the endothelial basement membrane, such as elastin and collagen [17]. This has been shown by a loss in integrity of the endothelial vascular barrier, resulting in increased permeability of the alveolar-capillary membrane [18]. Neutrophil elastase is thought to hydrolyse junction proteins such as cadherins, which maintain cell-cell adhesion and diminish barrier function. Similarly, it has been shown that NE can disrupt the epithelial barrier [19]. Taken together, NE can be responsible for protein leakage from the blood stream to the alveolus and vice versa.

Initially, NE was mostly used as a biomarker for the activation of PMNs* in vivo* after cardiopulmonary bypass [20], since extracorporeal circulation activates the complement system which in turn activates the PMNs [21].

A positive correlation has been observed between NE plasma concentrations after CPB and postoperative respiratory function, by changes in the respiratory index and increases in the intrapulmonary shunt [22]. In another study a positive correlation was found between the NE plasma concentration and the alveolar-arterial oxygen gradient and pulmonary vascular resistance [23].

In addition NE has been used to study the effects of leukocyte depletion during cardiopulmonary bypass [24, 25], NE inhibitors [26], pump types [27], and biocompatibility of leukocyte and fat removal filters [28].

Although NE can be a valuable biomarker in assessing PMNs induced lung injury, it is still only a measure of PMNs activation and not a specific lung biomarker.

### 5.2. Myeloperoxidase

Myeloperoxidase (MPO) is a peroxidase enzyme stored in the azurophilic granules of PMNs. Its primary function is to kill microorganisms in PMNs by forming halide derived oxidants in the phagosome [29]. Ischemia during cardiopulmonary bypass results in endothelial activation upon reperfusion [30]. The activated endothelium and the expression of specific surface adhesion molecules promote adherence of phagocytes [31], upon which MPO will be released. MPO measured in blood is a marker for degranulation of PMNs in plasma and for the infiltration of PMNs in tissue [32]. Besides being a marker for PMN degranulation, MPO is often implicated in lung injury. Pulmonary tissue is often the target of activated PMNs when it is being reperfused; therefore MPO concentrations are thought to be a marker of pulmonary injury.

During CPB, pulmonary endothelial permeability correlated with postoperative serum concentrations of MPO, implicating neutrophils having a central role in the development of lung injury [33]. However, we and others have shown that MPO shows a steep increase right after the administration of heparin [34, 35]. This increase of MPO after heparin administration is explained by liberation of MPO bound to the vessel wall [36], which suggests that an increase in plasma MPO does not necessarily represent activation/degranulation of leukocytes. This would also implicate that MPO is of limited use as a biomarker for assessing lung injury after CPB, which requires high dose heparin anticoagulation.

## 6. Lung Epithelium Specific Proteins

### 6.1. Soluble Receptor for Advanced Glycation End Products (sRAGE)

The receptor for advanced glycation end products (RAGE) was originally characterized for its ability to bind glycation end products of a carbohydrate to a protein. Besides advanced glycation end products, RAGE has the ability to bind several other ligands, for example, amphoterins, S100 proteins, Mac-1, phosphatidylserine, and complement C3a [37]. Expression of RAGE is encountered on multiple cell types such as smooth muscle cells, macrophages, and endothelial cells, but it is also highly expressed in the alveolar type I cells (Figure 1) of the lungs [38].

Soluble forms of RAGE can be formed by proteolytic cleavage of full length RAGE by metalloproteinases or by formation of a splice variant and can be measured in the blood stream [39]. Accordingly, this led to the potential application of (s)RAGE as a lung injury marker of alveolar type I cells. The function of circulating sRAGE is being investigated in various (clinical) studies and is not yet completely elucidated. However, sRAGE is thought to contribute to the removal and/or detoxification of proinflammatory products [39].

Indeed it has been shown that sRAGE is an injury marker of alveolar type I cells [40]. Uchida et al. found in patients with ALI that plasma concentrations were significantly higher than in patients with hydrostatic pulmonary edema or in healthy controls. Increased sRAGE plasma concentrations have also been associated with the use of CPB and mechanical ventilation in patients undergoing elective coronary artery bypass grafting [41]. More recently, Tuinman et al. showed that sRAGE plasma concentrations increased following valvular and/or coronary artery surgery and that they depicted an association with pulmonary leak index, indicating increased permeability of the alveolar-capillary membrane [42]. In young children, plasma concentrations of sRAGE were found to be an independent predictor of ALI after cardiac surgery with CPB [43]. Furthermore, in children as well as in adults increased plasma concentrations of sRAGE were associated with lower PaO_2_/FiO_2_ ratio, a higher radiographic lung injury score, longer mechanical ventilation time, and longer intensive care unit length of stay [43, 44].

Preoperative measurements of sRAGE are also of value. In a study where patients underwent elective cardiac surgery, preoperative sRAGE plasma concentrations were associated with duration of critical illness and length of hospital stay [45]. Furthermore, sRAGE was found to be an independent predictor of length of hospital stay.

Calfee et al. showed that sRAGE plasma concentrations measured four hours after allograft reperfusion were associated with poor short term outcome of lung transplantation, as indicated by longer duration of mechanical ventilation and longer intensive care unit length of stay [46]. This finding was supported by another study where an association was found between plasma concentrations of sRAGE and primary graft dysfunction at 6 and 24 hours following lung transplantation [47]. Furthermore an association between sRAGE plasma concentrations measured at 6 and 24 hours following transplantation and mechanical ventilation time was found. The same authors also established an association between sRAGE plasma levels measured at both 6 and 24 h postoperatively with long-term risk for bronchiolitis obliterans syndrome [48].

### 6.2. Clara Cell Secretory Protein

Clara cells are secretory epithelial cells lining the pulmonary airways (Figure 1). The exact role of these cells still remains unclear, although they are implicated in having a role in protecting and repairing the bronchial epithelium [49]. Clara cells are mainly located in the respiratory bronchioles and they have granules containing various proteins. One of these secretory proteins is Clara cell 16 kD secretory protein (CC16), which is referred to in literature by various names such as uteroglobin (UG), blastokinin, Clara cell secretory protein (CCSP), Clara cell-specific 10 kD protein (CC10), and secretoglobin 1A member 1 (SCGB1A1).

Clara cell secretory protein is believed to play a role in reducing inflammation of the airways [50] and protecting the respiratory tract against oxidative stress [51]. It is present in increasing density from the trachea to terminal bronchioles. Although there is evidence of extrapulmonary synthesis of the CC16 in the prostate, endometrium, and the kidney, these concentrations are on average twenty times lower than in the lungs [52]. This is the reason why CC16 is primarily ascribed to the respiratory tract and why it is considered to be lung specific.

With stable baseline serum concentrations of 10–20 ng/mL, an increase in serum is ascribed to injury to the alveolar-capillary membrane. When the membrane is known to be intact it could be related to the integrity of the Clara cell or the production and clearance of CC16.

Serum concentrations of CC16 have been associated with injury of the alveolar-capillary membrane and are nowadays often used as a biomarker of injury to the alveolar-capillary membrane in different models, such as ALI/ARDS [53, 54], cardiogenic pulmonary edema [54], chest trauma [55], chronic obstructive pulmonary disease [56, 57], primary graft dysfunction (PGD) [58], and injury due to fire exposure [59].

However, in the setting of cardiothoracic surgery CC16 has not often been used as a lung injury marker. Serum CC16 concentrations have been associated with bronchiolitis obliterans syndrome after lung transplantation [60] and primary graft dysfunction after lung transplantation [58]. In a more recent study, the same authors showed that even higher preoperative serum CC16 concentrations, measured in the recipient, were associated with primary graft dysfunction after lung transplantation [61]. Additionally, CC16 has been utilized as a lung injury marker for comparison of mechanical ventilation strategies during various surgical procedures [53], for comparison of a mini-extracorporeal circuit versus a conventional cardiopulmonary bypass [62–64], and for evaluation of pulsatile flow during CPB on lung function [65]. More recently, we have shown that CC16 concentrations correlate with pulmonary dysfunction (as indicated by the alveolar-arterial oxygen gradient) during cardiothoracic surgery and that it was possible to differentiate between off-pump and on-pump coronary artery bypass grafting [34]. In our opinion, given its small size which facilitates diffusion into the blood, this is a sensitive and very useful marker for detecting subclinical injury to the alveolar-capillary membrane.

### 6.3. Surfactant Proteins

Pulmonary surfactant is the main fraction of the epithelial lining fluid in the lungs. Its main function is to lower surface tension between air and the alveoli and thereby to prevent alveolar atelectasis at the end of expiration [66]. Pulmonary surfactant consists of lipids (90%) and proteins (5–10%). Type II alveolar epithelial cells are mainly responsible for synthesis and secretion of pulmonary surfactant (Figure 1), and before surfactant is secreted it is stored in organelles called “lamellar bodies.” The lipids of surfactant are mainly phospholipids, with phosphatidylcholine being the most abundant. Saturated phosphatidylcholine largely consists of dipalmitoylphosphatidylcholine (DPPC), which accounts for approximately 40% of total lipids and is the major surface-active component.

The protein fraction of surfactant is of more interest for this review. This fraction consists of four different surfactant proteins, SP-A, SP-B, SP-C, and SP-D. Surfactant proteins B (14 kDa) and C (6 kDa) are hydrophobic and are involved in phospholipid packaging, organization of surfactant, and lowering the surface tension at the air-liquid interface [67, 68]. Via its interaction with DPPC, SP-B has been considered to stabilize the phospholipid monolayer. Similarly, SP-C is also thought to be involved in stabilizing the phospholipid layers that form during film compression at low lung volumes [69].

The hydrophilic surfactant proteins, SP-A and SP-D, are predominantly involved in the innate host-defence system of the lung [70] and belong to the collectin family (along with mannose-binding lectin). They are assembled as a trimeric structure with the carbohydrate recognition domain connected to a collagenous domain [71]. The carbohydrate recognition domain has a high affinity for clustered oligosaccharides commonly found on the surface of viruses, bacteria, yeast, and fungi, which can lead to agglutination, phagocytosis, and removal by macrophages and neutrophils or by direct bacteriostatic and fungistatic effects [72].

So far, the use of surfactant protein leakage in blood during cardiothoracic surgery is limited. Agostoni et al. evaluated SP-B as a lung injury marker after elective coronary artery bypass grafting with the use of cardiopulmonary bypass [41]. Immediately after surgery, they found a fourfold increase of plasma SP-B, which returned to baseline within 48 hours. The authors concluded that SP-B could be a sensitive and rapid biomarker of lung distress. Unfortunately, due to small sample size and relatively healthy patients, they could not relate the change in SP-B to severity of lung injury. The same group has, however, shown that plasma SP-B levels are related to alveolar gas diffusion showing a link between SP-B plasma levels and injury to the alveolar-capillary membrane [73].

Sims et al. evaluated the use of SP-D as a lung injury marker in patients undergoing lung transplantation [74]. They found that SP-D serum concentrations were higher in idiopathic pulmonary fibrosis than in cystic fibrosis, chronic obstructive pulmonary disease, or pulmonary hypertension. During transplantation they found that SP-D concentrations decreased. However, postoperative values were higher in single lung transplantation as opposed to the bilateral lung transplantation. The authors suggested that postoperative SP-D concentrations were more likely to be determined by the inflamed native lung as opposed to the allograft, leaving the native lung as the source for SP-D translocation.

Determann et al. have used circulating plasma concentrations of SP-A and SP-D to evaluate mechanical ventilation strategies where a lower tidal volume was used [75]. They did not find differences in plasma concentrations of these surfactant proteins, which was consistent with clinical data as none of the patients showed signs of advanced lung injury.

Shah et al. used plasma SP-D, among other biomarkers, to better discriminate clinically graded primary graft dysfunction and to predict 90-day mortality after lung transplantation [76]. They found that SP-D together with plasminogen activator inhibitor-1 plasma concentrations, measured 24 h after transplantation, had an area under the curve of 0.76 for predicting grade 3 PGD in the first 72 h after transplantation. Furthermore, SP-D significantly increased prediction over PGD grading alone in 90-day mortality, although the other evaluated biomarkers performed even better.

In our experience SP-D can be a valuable marker during cardiothoracic surgery: we found that SP-D concentrations correlated with pulmonary (dys) function and that it was possible to differentiate surgical procedures (off-pump versus on-pump) [34].

### 6.4. Krebs von den Lungen 6

Krebs von den Lungen 6 (KL-6) is a mucinous sialylated sugar chain on human Mucin 1 [77]. Mucin 1 is a transmembrane protein with an extracellular domain, containing tandem repeat units that are heavily glycosylated. Mucins line the apical surface of epithelial cells in the bronchi, bronchioles, and alveoli where KL-6 is mainly expressed on alveolar type II cells [78] and expression is upregulated on regenerating alveolar type II cells [79]. KL-6 can be found in bronchoalveolar lavage fluid or in serum, and concentrations are elevated in patients with interstitial lung diseases, such as pulmonary sarcoidosis [80, 81]. Additionally, KL-6 seems to be a valuable biomarker in diagnosing bronchiolitis obliterans syndrome after lung transplantation [82, 83].

Although KL-6 plasma concentrations have been used as a marker of disease activity in a variety of respiratory illnesses, the use of this marker in cardiothoracic surgery remains limited. There is one report where it has been used for comparing between a mini cardiopulmonary bypass system and a conventional bypass system, but it failed to detect a difference between the two systems [63].

## 7. Inflammatory Secretion Products

The inflammatory secretion products discussed here are produced by a broad range of cell types; however in the alveoli the macrophage is one of the major sources. Alveolar macrophages are located at the luminal interface of the alveoli or in the interstitium and remove (dust) particles and/or microorganisms. Since the lungs are in contact with the outer world, they are exposed to a vast array of pathogens, chemicals, gasses, and particles. Besides a mucociliary layer for removal of these substances, alveolar macrophages are important for “cleaning” and defending the alveolar-capillary membrane. Upon activation, macrophages remove pathogens and foreign substances by phagocytosis and simultaneously secrete mediators of inflammation and complement proteins. Activated macrophages can be divided by activation state; these are known as M1 (or classically activated macrophages) and M2 (or alternatively activated macrophages) [84, 85]. While the M1 macrophages promote inflammation, extracellular matrix destruction, and apoptosis, the M2 macrophages promote extracellular matrix construction, cell proliferation, and angiogenesis. These two types of activation come with their own characteristic secretory profile of (anti)inflammatory cytokines, chemokines, and proteolytic enzymes. On the one hand, M1 macrophages will release proinflammatory cytokines, such as IL-1*β*, IL-6, and TNF-*α* [86, 87]. Additionally, the chemokines IL-8, IL-10, MIP-1*α*, and MIP-1*β* and the matrix metalloproteases 1, 2, 7, 8, and 12 are released [88], which can degrade collagen, elastin, fibronectin, and other extracellular matrix components. On the other hand, M2 macrophages will release chemokines CCL17, CCL18, and CCL22 along with the anti-inflammatory cytokines Il-10 and TGF-*β* [89, 90].

In the setting of cardiothoracic surgery, with its more acute characteristics, the secretory products of the M1 macrophages are most interesting for assessing lung injury. During cardiothoracic surgery the lungs experience ischemia/reperfusion when cardiopulmonary bypass is used. Ischemia/reperfusion is known to be a strong stimulus to M1 macrophages [91, 92], upon which the aforementioned proinflammatory substances are released. These proinflammatory substances could be valuable predicting biomarkers for lung injury and/or lung dysfunction. And indeed, proteomic analysis showed that isolated alveolar macrophages, harvested during the course of ALI/ARDS, had an upregulated inflammatory profile [93]. Amongst these upregulated proteins was cathepsin B, a lysosomal cysteine proteinase, which the authors suggested to be a biomarker for early diagnoses of ALI/ARDS. During cardiothoracic surgery, however, this protein has not yet been used as a biomarker for lung injury.

Monocyte chemotactic protein 1 (CCL2), primarily secreted by monocytes, macrophages, and dendritic cells, was associated with complicated inflammatory lung or renal injury in patients undergoing primary elective coronary artery bypass grafting [94]. During lung transplantation, MCP-1 and interferon gamma-induced protein 10 (IP-10) were associated with the development of primary graft dysfunction, and from 6 to 72 hours following transplantation MCP-1 and IP-10 concentrations were significantly higher in patients with primary graft dysfunction [95]. In another lung transplantation study it was shown that interleukins 6, 8, and 10 were also associated with primary graft dysfunction [96], where IL-10 peaked at the start of reperfusion and IL-6 and IL-8 peaked 4 hours after transplantation.

In patients with moderate chronic obstructive pulmonary disease undergoing aortic valve surgery, leukocyte filtration during CPB resulted in lower plasma concentrations of IL-6, IL-8, and TNF-*α* from CPB discontinuation till 72 h postoperatively [97]. Furthermore, the authors found a linear correlation between IL-6 and TNF-*α* with the alveolar-arterial oxygen gradient (Aa-O_2_ gradient) and an inverse linear correlation between IL-6 and IL-8 with the arterial blood oxygenation (PaO_2_/FiO_2_).

## 8. Limitations

Besides the advantages described earlier, there are some limitations to the use of biomarkers. One limitation is the high variability in measuring biomarkers, which makes comparing studies more difficult. Most of the variability can be attributed to preanalytical and/or analytical variability [98], where preanalytical variability refers to stability over time and biological variability (e.g., age, sex, and ethnicity), and analytical variability refers to the performance of the test in the laboratory (validity, sensitivity, specificity, and reproducibility, amongst others). The high analytical variability is illustrated by a study where factors influencing the measurement of plasma SP-D by ELISA were examined [99]. It was found that the ELISA configuration (different manufactures) and the anticoagulant used could have serious effects on the measured SP-D concentration. For instance, the use of EDTA instead of heparin reduced the measured SP-D concentration by 50%.

Timing is critical when measuring biomarkers. Most biomarkers reviewed here show a postoperative increase. However, this increase can be of short duration, preventing possible detection when the time points for sampling are not optimally chosen. For instance, we studied SP-D and CC16 in patients undergoing either on- or off-pump coronary artery bypass grafting [34]. The largest difference between these two groups was at the end of CPB, while one hour after arrival on the intensive care unit the difference between these biomarkers was no longer significant. Having to sample many time points limits the cost effectiveness of biomarkers.

Failure to identify factors that can influence the measurement of a biomarker can lead to confounding effects. These effects can be patient characteristics, such as age, sex, weight, and use of medication, although groups are usually balanced for these potential confounding effects. An effect which, for instance, is often overlooked is the stability of a biomarker when it is stored for a prolonged period of time. When the inclusion of a study takes months or even years and the biomarker degrades when it is stored, large differences in measured biomarker concentrations between the first included patient and the last included patient can occur.

## 9. Concluding Remarks

In this review, we discussed different biomarkers to identify lung injury after cardiothoracic surgery, most often with the use of cardiopulmonary bypass. Though many biomarkers for lung injury are known, they are not often incorporated in clinical studies. For the several good biomarkers available for quantifying lung injury after cardiothoracic surgery, the clinical applications are significant. They enable early detection of patients with subtle injury when they are adequately sensitive and specific. In addition, it would assist in the development of improved surgical techniques to prevent injury after cardiothoracic surgery. For this purpose a panel of biomarkers is most informative, especially when biomarkers for alveolar types I and II cell injury are incorporated.
